# *N*-(2,4-Dinitro­phen­yl)-*N*′-[nitro­(phenyl)­methyl­ene]hydrazine

**DOI:** 10.1107/S1600536808036179

**Published:** 2008-11-08

**Authors:** Chunlan Yuan

**Affiliations:** a Department of Chemistry and Chemical Engineering, Baoji College of Arts and Sciences, Baoji 721007, People’s Republic of China

## Abstract

The title compound, C_13_H_9_N_5_O_6_, contains three nitro groups. It is prepared by the reaction of benzaldehyde 2,4-dinitro­phenyl­hydrazone with nitric oxide at ambient temperature. The imine group is nearly coplanar with the (2,4-dinitro­phen­yl)­hydrazine unit. The second benzene ring and the third nitro group are twisted away from this plane, with dihedral angles of 48.5 (3) and 15.2 (3)°, respectively. Weak intra­molecular N—H⋯O inter­actions are observed.

## Related literature

For related literature regarding NO, see: Garthwaite *et al.* (1989 ▶); Murad (1999 ▶). For aryl­hydrazones, see: Chan *et al.* (2001 ▶); Försterling & Barnes (2001 ▶); Paschalidis *et al.* (2000 ▶). For the structure of benzaldehyde 2,4-dinitro­phenyl­hydrazone, see Shan *et al.* (2003 ▶).
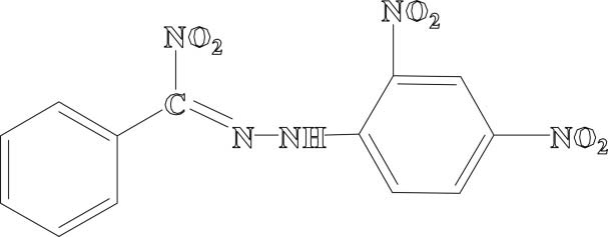


## Experimental

### 

#### Crystal data

C_13_H_9_N_5_O_6_
*M* *_r_* = 331.25
Orthorhombic, 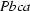

*a* = 6.9790 (1) Å
*b* = 13.469 (2) Å
*c* = 29.448 (8) Å
*V* = 2768.1 (9) Å^3^
*Z* = 8
Mo *K*α radiation
μ = 0.13 mm^−1^
*T* = 289 (2) K
0.52 × 0.48 × 0.22 mm

#### Data collection

Siemens P4 diffractometer
Absorption correction: none
3591 measured reflections
3018 independent reflections
1537 reflections with *I* > 2σ(*I*)
*R*_int_ = 0.0000
3 standard reflections every 97 reflections intensity decay: 1.0%

#### Refinement

*R*[*F* ^2^ > 2σ(*F* ^2^)] = 0.039
*wR*(*F* ^2^) = 0.059
*S* = 0.98
3018 reflections
222 parameters
H atoms treated by a mixture of independent and constrained refinement
Δρ_max_ = 0.20 e Å^−3^
Δρ_min_ = −0.14 e Å^−3^

### 

Data collection: *XSCANS* (Siemens, 1996 ▶); cell refinement: *XSCANS*; data reduction: *SHELXTL* (Sheldrick, 2008 ▶); program(s) used to solve structure: *SHELXS97* (Sheldrick, 2008 ▶); program(s) used to refine structure: *SHELXL97* (Sheldrick, 2008 ▶); molecular graphics: *SHELXTL*; software used to prepare material for publication: *SHELXTL*.

## Supplementary Material

Crystal structure: contains datablocks I, New_Global_Publ_Block. DOI: 10.1107/S1600536808036179/ez2141sup1.cif

Structure factors: contains datablocks I. DOI: 10.1107/S1600536808036179/ez2141Isup2.hkl

Additional supplementary materials:  crystallographic information; 3D view; checkCIF report

## Figures and Tables

**Table 1:** Hydrogen-bond geometry (Å, °)

*D*—H⋯*A*	*D*—H	H⋯*A*	*D*⋯*A*	*D*—H⋯*A*
N2—H2N⋯O2	0.894 (15)	1.966 (15)	2.591 (2)	125.7 (13)

**Fig. 1.**
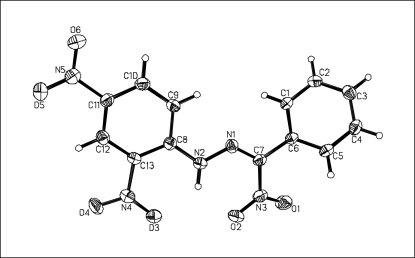
The structure of the dimer formation in (I),showing the atom-numbering scheme. Displacement ellipsoids are drawn at the 30% probability level and H atoms are not shown.

**Fig. 2.**
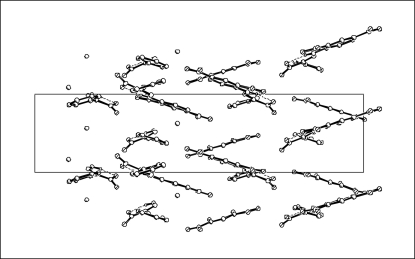
The crystal packing of (I), viewed along the b axis.

## supplementary crystallographic information

### Comment

Nitric oxide (NO) has been found recently to play an important role in chemistry, biology and medicine (Garthwaite *et al.*, 1989; Murad, 1999). In recent years, arylhydrazones have been utilized for the analysis of carbonyl compounds (Chan *et al.*, 2001). Some arylhydrazones and their nitration products were found to have pharmacological properties (Försterling & Barnes, 2001; Paschalidis *et al.*, 2000). Here we report the reaction of NO with an arylhydrazone, where the title compound, (I), was obtained by the reaction of NO with benzaldehyde-2,4-dinitrophenylhydrazone.

The structure of (I) (Fig.1), shows that this reaction resulted in the addition of a third NO_2_ group, which is attached to the carbon atom C7, with an O1—N3—C7—C6 torsion angle of -11.6 (3)°. The imine double bond in benzaldehyde 2,4-dinitrophenylhydrazone was preserved, as indicated by the N1═C7 distance [1.2779 (19) Å] being similar to that of 1.275 (2)Å in the original compound (Shan *et al.*, 2003). The other two nitro groups are co-planar with the benzene ring that they are attached to, with O4—N4—C12—C13 and O5—N5—C11—C12 torsion angles of 7.0 (3) and 0.0 (3)° respectively. There is a weak intramolecular N2—H(2 N)···O2 interaction.

### Experimental

A stock solution was prepared by dissolving 0.5 mol benzaldehyde -2,4-dinitrophenylhydrazine in 100 ml dry CH_2_Cl_2_. NO was produced by the reaction of 1 *M* H_2_SO_4_ solution trickled into an aqueous saturated NaNO_2_ solution through a funnel at a pre-determined speed, while stirring under an argon atmosphere. NO was carried by argon and purified by passing it through a series of scrubbing bottles containing 4*M* NaOH, distilled water and CaCl_2_ in turn. All the above bottles were under an argon atmosphere. The purified NO was bubbled through a previously degassed stirred stock solution at room temperature for an appropriate time. After the reaction was completed, as indicated by TLC, the reaction mixture was dried with anhydrous MgSO_4_, concentrated under vacuum and purified by column chromatography on silica–gel (200–300 mesh, ethyl acetate–hexane) yielding the pure title compound.

### Refinement

Atom H2N was located in a difference Fourier map and refined isotropically, with the N—H distance restrained to 0.89 Å. Other H atoms were placed in idealized positions and constrained to ride on their parent atoms, with C—H distances of 0.93Å and with *U*_iso_(H)= 1.2*U*_eq_(C,*N*).

### Crystal data

**Table:**

C_13_H_9_N_5_O_6_	*D*_x_ = 1.590 Mg m^−^^3^
*M**_r_* = 331.25	Mo *K*α radiation λ = 0.71073 Å
Orthorhombic, *P**b**c**a*	Cell parameters from 26 reflections
*a* = 6.9790 (1) Å	θ = 3.4–12.5º
*b* = 13.469 (2) Å	µ = 0.13 mm^−^^1^
*c* = 29.448 (8) Å	*T* = 289 (2) K
*V* = 2768.1 (9) Å^3^	Prism, yellow
*Z* = 8	0.52 × 0.48 × 0.22 mm
*F*_000_ = 1360	

### Data collection

**Table:**

Siemens P4 diffractometer	*R*_int_ = 0.0000
Radiation source: normal-focus sealed tube	θ_max_ = 27.0º
Monochromator: graphite	θ_min_ = 1.4º
*T* = 289(2) K	*h* = 0→8
ω scans	*k* = 0→17
Absorption correction: none	*l* = 0→37
3591 measured reflections	3 standard reflections
3018 independent reflections	every 97 reflections
1537 reflections with *I* > 2σ(*I*)	intensity decay: 1.0%

### Refinement

**Table:**

Refinement on *F*^2^	Hydrogen site location: inferred from neighbouring sites
Least-squares matrix: full	H atoms treated by a mixture of independent and constrained refinement
*R*[*F*^2^ > 2σ(*F*^2^)] = 0.039	*w* = 1/[σ^2^(*F*_o_^2^) + (0.0116*P*)^2^] where *P* = (*F*_o_^2^ + 2*F*_c_^2^)/3
*wR*(*F*^2^) = 0.059	(Δ/σ)_max_ = 0.001
*S* = 0.98	Δρ_max_ = 0.20 e Å^−^^3^
3018 reflections	Δρ_min_ = −0.14 e Å^−^^3^
222 parameters	Extinction correction: SHELXL, Fc^*^=kFc[1+0.001xFc^2^λ^3^/sin(2θ)]^-1/4^
Primary atom site location: structure-invariant direct methods	Extinction coefficient: 0.00375 (18)
Secondary atom site location: difference Fourier map	

### Special details

**Table:**

Geometry. All e.s.d.'s (except the e.s.d. in the dihedral angle between two l.s. planes) are estimated using the full covariance matrix. The cell e.s.d.'s are taken into account individually in the estimation of e.s.d.'s in distances, angles and torsion angles; correlations between e.s.d.'s in cell parameters are only used when they are defined by crystal symmetry. An approximate (isotropic) treatment of cell e.s.d.'s is used for estimating e.s.d.'s involving l.s. planes.
Refinement. Refinement of *F*^2^ against ALL reflections. The weighted *R*-factor *wR* and goodness of fit *S* are based on *F*^2^, conventional *R*-factors *R* are based on *F*, with *F* set to zero for negative *F*^2^. The threshold expression of *F*^2^ > σ(*F*^2^) is used only for calculating *R*-factors(gt) *etc*. and is not relevant to the choice of reflections for refinement. *R*-factors based on *F*^2^ are statistically about twice as large as those based on *F*, and *R*- factors based on ALL data will be even larger.

### Fractional atomic coordinates and isotropic or equivalent isotropic displacement parameters (Å^2^)

**Table:**

	*x*	*y*	*z*	*U*_iso_*/*U*_eq_	
O1	0.2792 (2)	0.72703 (10)	0.26225 (4)	0.0655 (5)	
O2	0.4128 (2)	0.58358 (10)	0.27056 (4)	0.0696 (5)	
O3	0.5250 (2)	0.37214 (9)	0.30832 (4)	0.0587 (4)	
O4	0.5513 (2)	0.23410 (9)	0.34501 (4)	0.0668 (5)	
O5	0.7770 (3)	0.22039 (11)	0.49622 (4)	0.0826 (6)	
O6	0.8082 (2)	0.35414 (10)	0.53534 (4)	0.0827 (6)	
N1	0.5194 (2)	0.63895 (10)	0.35787 (5)	0.0411 (4)	
N2	0.5362 (2)	0.54088 (11)	0.35121 (5)	0.0423 (4)	
N3	0.3764 (3)	0.66797 (13)	0.28337 (5)	0.0489 (5)	
N4	0.5574 (2)	0.32486 (12)	0.34320 (5)	0.0463 (4)	
N5	0.7673 (3)	0.31066 (14)	0.50021 (5)	0.0599 (5)	
C1	0.3799 (3)	0.82777 (13)	0.38520 (6)	0.0409 (5)	
H1	0.3459	0.7770	0.4050	0.049*	
C2	0.3749 (3)	0.92498 (14)	0.39969 (6)	0.0459 (5)	
H2	0.3372	0.9394	0.4293	0.055*	
C3	0.4251 (3)	1.00085 (14)	0.37089 (6)	0.0490 (6)	
H3	0.4223	1.0663	0.3810	0.059*	
C4	0.4797 (3)	0.97942 (14)	0.32689 (6)	0.0509 (6)	
H4	0.5137	1.0307	0.3073	0.061*	
C5	0.4842 (3)	0.88204 (14)	0.31166 (6)	0.0449 (5)	
H5	0.5195	0.8682	0.2819	0.054*	
C6	0.4359 (3)	0.80519 (13)	0.34096 (6)	0.0368 (5)	
C7	0.4514 (3)	0.69945 (13)	0.32858 (6)	0.0387 (5)	
C8	0.5928 (3)	0.48344 (13)	0.38722 (6)	0.0376 (5)	
C9	0.6383 (3)	0.52778 (13)	0.42912 (6)	0.0433 (5)	
H9	0.6295	0.5964	0.4322	0.052*	
C10	0.6953 (3)	0.47186 (14)	0.46549 (6)	0.0453 (5)	
H10	0.7271	0.5023	0.4928	0.054*	
C11	0.7052 (3)	0.37003 (14)	0.46136 (6)	0.0424 (5)	
C12	0.6595 (3)	0.32304 (13)	0.42157 (6)	0.0422 (5)	
H12	0.6654	0.2542	0.4194	0.051*	
C13	0.6048 (3)	0.37961 (13)	0.38483 (6)	0.0376 (5)	
H2N	0.508 (2)	0.5131 (11)	0.3244 (5)	0.045 (6)*	

### Atomic displacement parameters (Å^2^)

**Table:**

	*U*^11^	*U*^22^	*U*^33^	*U*^12^	*U*^13^	*U*^23^
O1	0.0715 (11)	0.0759 (10)	0.0491 (9)	0.0080 (10)	−0.0181 (8)	−0.0017 (8)
O2	0.1089 (14)	0.0566 (9)	0.0433 (8)	0.0080 (10)	−0.0062 (9)	−0.0146 (7)
O3	0.0786 (12)	0.0550 (9)	0.0426 (8)	−0.0004 (9)	−0.0118 (8)	−0.0065 (7)
O4	0.0932 (13)	0.0360 (8)	0.0711 (10)	0.0018 (9)	−0.0160 (9)	−0.0131 (7)
O5	0.1295 (16)	0.0517 (10)	0.0666 (10)	0.0200 (12)	−0.0158 (11)	0.0040 (9)
O6	0.1324 (17)	0.0721 (11)	0.0437 (8)	0.0087 (11)	−0.0200 (10)	−0.0044 (8)
N1	0.0411 (11)	0.0381 (9)	0.0443 (9)	0.0011 (9)	0.0036 (8)	0.0007 (8)
N2	0.0489 (12)	0.0416 (10)	0.0365 (10)	−0.0013 (9)	−0.0036 (9)	−0.0058 (8)
N3	0.0519 (13)	0.0583 (12)	0.0366 (10)	−0.0064 (11)	0.0015 (9)	0.0009 (9)
N4	0.0429 (12)	0.0467 (11)	0.0492 (10)	0.0015 (9)	−0.0012 (10)	−0.0086 (9)
N5	0.0738 (15)	0.0586 (13)	0.0471 (11)	0.0100 (12)	−0.0023 (11)	0.0012 (10)
C1	0.0408 (13)	0.0460 (12)	0.0358 (11)	0.0008 (11)	−0.0005 (10)	0.0064 (9)
C2	0.0467 (13)	0.0548 (13)	0.0362 (11)	0.0046 (12)	0.0001 (10)	−0.0056 (10)
C3	0.0483 (14)	0.0410 (12)	0.0577 (13)	0.0047 (11)	−0.0079 (12)	−0.0038 (11)
C4	0.0557 (15)	0.0493 (13)	0.0478 (12)	−0.0006 (12)	−0.0034 (12)	0.0138 (10)
C5	0.0477 (14)	0.0556 (13)	0.0315 (10)	0.0000 (11)	−0.0016 (10)	0.0050 (10)
C6	0.0350 (12)	0.0441 (11)	0.0312 (10)	0.0009 (10)	−0.0025 (9)	0.0009 (9)
C7	0.0397 (13)	0.0449 (12)	0.0314 (10)	−0.0033 (11)	0.0025 (10)	−0.0002 (9)
C8	0.0357 (12)	0.0393 (11)	0.0377 (11)	−0.0021 (10)	0.0033 (10)	0.0007 (9)
C9	0.0518 (14)	0.0379 (11)	0.0404 (11)	0.0013 (11)	0.0042 (10)	−0.0063 (9)
C10	0.0509 (15)	0.0497 (12)	0.0352 (11)	0.0000 (12)	0.0022 (10)	−0.0045 (10)
C11	0.0462 (14)	0.0458 (12)	0.0352 (10)	0.0029 (12)	0.0016 (10)	0.0022 (10)
C12	0.0402 (12)	0.0377 (11)	0.0488 (12)	0.0020 (10)	0.0046 (10)	−0.0015 (10)
C13	0.0362 (12)	0.0398 (11)	0.0367 (10)	−0.0014 (10)	0.0006 (10)	−0.0080 (9)

### Geometric parameters (Å, °)

**Table:**

O1—N3	1.2163 (17)	C2—H2	0.9300
O2—N3	1.2244 (17)	C3—C4	1.381 (2)
O3—N4	1.2296 (17)	C3—H3	0.9300
O4—N4	1.2244 (17)	C4—C5	1.386 (2)
O5—N5	1.2233 (17)	C4—H4	0.9300
O6—N5	1.2227 (18)	C5—C6	1.389 (2)
N1—C7	1.2779 (19)	C5—H5	0.9300
N1—N2	1.3405 (18)	C6—C7	1.474 (2)
N2—C8	1.371 (2)	C8—C13	1.403 (2)
N2—H2N	0.894 (15)	C8—C9	1.407 (2)
N3—C7	1.492 (2)	C9—C10	1.368 (2)
N4—C13	1.468 (2)	C9—H9	0.9300
N5—C11	1.462 (2)	C10—C11	1.379 (2)
C1—C2	1.378 (2)	C10—H10	0.9300
C1—C6	1.394 (2)	C11—C12	1.369 (2)
C1—H1	0.9300	C12—C13	1.377 (2)
C2—C3	1.373 (2)	C12—H12	0.9300
			
C7—N1—N2	124.20 (15)	C4—C5—H5	120.0
N1—N2—C8	117.91 (15)	C6—C5—H5	120.0
N1—N2—H2N	121.5 (10)	C5—C6—C1	119.08 (16)
C8—N2—H2N	120.6 (10)	C5—C6—C7	123.26 (16)
O1—N3—O2	124.43 (17)	C1—C6—C7	117.56 (16)
O1—N3—C7	117.78 (16)	N1—C7—C6	118.45 (16)
O2—N3—C7	117.76 (17)	N1—C7—N3	123.46 (16)
O4—N4—O3	123.17 (16)	C6—C7—N3	117.99 (16)
O4—N4—C13	118.22 (16)	N2—C8—C13	122.75 (17)
O3—N4—C13	118.61 (15)	N2—C8—C9	120.25 (16)
O6—N5—O5	122.99 (18)	C13—C8—C9	116.98 (17)
O6—N5—C11	118.00 (17)	C10—C9—C8	121.21 (17)
O5—N5—C11	119.01 (17)	C10—C9—H9	119.4
C2—C1—C6	120.27 (17)	C8—C9—H9	119.4
C2—C1—H1	119.9	C9—C10—C11	119.55 (17)
C6—C1—H1	119.9	C9—C10—H10	120.2
C3—C2—C1	120.63 (17)	C11—C10—H10	120.2
C3—C2—H2	119.7	C12—C11—C10	121.58 (18)
C1—C2—H2	119.7	C12—C11—N5	119.07 (17)
C2—C3—C4	119.61 (17)	C10—C11—N5	119.35 (17)
C2—C3—H3	120.2	C11—C12—C13	118.75 (17)
C4—C3—H3	120.2	C11—C12—H12	120.6
C3—C4—C5	120.49 (18)	C13—C12—H12	120.6
C3—C4—H4	119.8	C12—C13—C8	121.91 (17)
C5—C4—H4	119.8	C12—C13—N4	116.14 (16)
C4—C5—C6	119.90 (17)	C8—C13—N4	121.95 (17)
			
C7—N1—N2—C8	−173.30 (18)	N2—C8—C9—C10	−179.95 (17)
C6—C1—C2—C3	−0.1 (3)	C13—C8—C9—C10	1.3 (3)
C1—C2—C3—C4	0.5 (3)	C8—C9—C10—C11	−1.2 (3)
C2—C3—C4—C5	0.0 (3)	C9—C10—C11—C12	0.1 (3)
C3—C4—C5—C6	−0.8 (3)	C9—C10—C11—N5	179.60 (17)
C4—C5—C6—C1	1.2 (3)	O6—N5—C11—C12	179.67 (19)
C4—C5—C6—C7	−175.08 (19)	O5—N5—C11—C12	0.0 (3)
C2—C1—C6—C5	−0.7 (3)	O6—N5—C11—C10	0.2 (3)
C2—C1—C6—C7	175.76 (18)	O5—N5—C11—C10	−179.5 (2)
N2—N1—C7—C6	178.19 (17)	C10—C11—C12—C13	0.8 (3)
N2—N1—C7—N3	1.8 (3)	N5—C11—C12—C13	−178.71 (17)
C5—C6—C7—N1	137.61 (19)	C11—C12—C13—C8	−0.6 (3)
C1—C6—C7—N1	−38.7 (3)	C11—C12—C13—N4	−179.89 (16)
C5—C6—C7—N3	−45.8 (3)	N2—C8—C13—C12	−179.11 (17)
C1—C6—C7—N3	137.86 (16)	C9—C8—C13—C12	−0.4 (3)
O1—N3—C7—N1	164.76 (18)	N2—C8—C13—N4	0.1 (3)
O2—N3—C7—N1	−13.5 (3)	C9—C8—C13—N4	178.85 (16)
O1—N3—C7—C6	−11.6 (3)	O4—N4—C13—C12	7.0 (3)
O2—N3—C7—C6	170.13 (18)	O3—N4—C13—C12	−173.35 (17)
N1—N2—C8—C13	176.35 (17)	O4—N4—C13—C8	−172.31 (18)
N1—N2—C8—C9	−2.3 (3)	O3—N4—C13—C8	7.4 (3)

### Hydrogen-bond geometry (Å, °)

**Table:**

*D*—H···*A*	*D*—H	H···*A*	*D*···*A*	*D*—H···*A*
N2—H2N···O2	0.894 (15)	1.966 (15)	2.591 (2)	125.7 (13)
